# Immune checkpoint inhibitor therapy increases systemic SDF-1, cardiac DAMPs Fibronectin-EDA, S100/Calgranulin, galectine-3, and NLRP3-MyD88-chemokine pathways

**DOI:** 10.3389/fcvm.2022.930797

**Published:** 2022-09-08

**Authors:** Vincenzo Quagliariello, Margherita Passariello, Annabella Di Mauro, Ciro Cipullo, Andrea Paccone, Antonio Barbieri, Giuseppe Palma, Antonio Luciano, Simona Buccolo, Irma Bisceglia, Maria Laura Canale, Giuseppina Gallucci, Alessandro Inno, Claudia De Lorenzo, Nicola Maurea

**Affiliations:** ^1^Division of Cardiology, Istituto Nazionale Tumori- Istituto di Ricovero e Cura a Carattere Scientifico (IRCCS)- Fondazione G. Pascale, Naples, Italy; ^2^Department of Molecular Medicine and Medical Biotechnology, University of Naples “Federico II”, Naples, Italy; ^3^Ceinge-Biotecnologie Avanzate s.c.a.r.l., Naples, Italy; ^4^Pathology Unit, Istituto Nazionale Tumori- Istituto di Ricovero e Cura a Carattere Scientifico (IRCCS)- Fondazione G. Pascale, Naples, Italy; ^5^Animal Facility, Istituto Nazionale Tumori- Istituto di Ricovero e Cura a Carattere Scientifico (IRCCS)- Fondazione G. Pascale, Naples, Italy; ^6^Servizi Cardiologici Integrati, Dipartimento Cardio-Toraco-Vascolare, Azienda Ospedaliera San Camillo Forlanini, Rome, Italy; ^7^U.O.C. Cardiologia, Ospedale Versilia, Lido di Camaiore (LU), Camaiore, Italy; ^8^Cardiologia, Centro di Riferimento Oncologico della Basilicata (CROB) - Istituto di Ricovero e Cura a Carattere Scientifico (IRCCS), Rionero in Vulture, Italy; ^9^Medical Oncology, Istituto di Ricovero e Cura a Carattere Scientifico (IRCCS) Ospedale Sacro Cuore Don Calabria, Negrar, Italy

**Keywords:** immunotherapy, biomarkers, preclinical study, mechanisms, inflammation, interleukin

## Abstract

**Background:**

Immune checkpoint inhibitors (ICIs) have significantly changed the oncology clinic in recent years, improving survival expectations in cancer patients. ICI therapy have a broad spectrum of side effects from endocrinopathies to cardiovascular diseases. In this study, pro-inflammatory and pro-fibrotic effects of short-term ICIs therapy in preclinical models were analyzed.

**Methods:**

Firstly, in a human *in vitro* model, human cardiomyocytes co-cultured with hPBMC were exposed to ICIs (with CTLA-4 or PD-1 blocking agents, at 200 nM) for 72 h. After treatment, production of DAMPs and 12 cytokines were analyzed in the supernatant through colorimetric and enzymatic assays. C57/Bl6 mice were treated with CTLA-4 or PD-1 blocking agents (15 mg/kg) for 10 days. Before (T0), after three days (T3) and after treatments (T10), ejection fraction, fractional shortening, radial and longitudinal strain were calculated by using bidimensional echocardiography (Vevo 2100, Fujfilm). Fibrosis, necrosis, hypertrophy and vascular NF-kB expression were analyzed through Immunohistochemistry. Myocardial expression of DAMPs (S100- Calgranulin, Fibronectin and Galectine-3), MyD88, NLRP3 and twelve cytokines have been analyzed. Systemic levels of SDF-1, IL-1β, and IL-6 were analyzed before, during and after ICIs therapy.

**Results:**

Radial and longitudinal strain were decreased after 10 days of ICIs therapy. Histological analysis of NF-kB expression shows that short-term anti-CTLA-4 or anti-PD-1 treatment increased vascular and myocardial inflammation. No myocardial hypertrophy was seen with the exception of the pembrolizumab group. Myocardial fibrosis and expression of galectin-3, pro-collagen 1-α and MMP-9 were increased after treatment with all ICIs. Both anti-CTLA-4 or anti-PD-1 treatments increased the expression of DAMPs, NLRP3 inflammasome and MyD88 and induced both *in vitro* and *in vivo* the secretion of IL-1β, TNF-α and IL-6. Systemic levels of SDF-1, IL-1β and IL-6 were increased during and after treatment with ICIs.

**Conclusions:**

Short therapy with PD-1 and CTLA-4 blocking agents increases vascular expression of NF-kB, systemic SDF-1, IL-1β, IL-6 levels and myocardial NLRP3, MyD88 and DAMPs expression in preclinical models. A pro-inflammatory cytokine storm was induced in myocardial tissues and in cultured cardiac cells after ICIs therapy. The overall picture of the study suggests new putative biomarkers of ICIs-mediated systemic and myocardial damages potentially useful in clinical cardioncology.

## Introduction

Immune checkpoint inhibitors (ICIs) includes monoclonal antibodies that activate the host immune system for efficient killing of cancer cells through unspecific activation mechanisms (1). ICIs are directed against programmed cell death protein (PD-1), its associated ligand (PD-L1) or CTLA-4 **(**Cytotoxic T-Lymphocyte Antigen 4) leading to activation of lymphocytes and NK cell activity against cancer cells (2, 3). Clinical benefits were seen in melanoma, non-small cell lung cancer and metastatic breast cancer patients; association therapies with radiotherapy or chemotherapy or with other ICIs are still proposed worldwide (2). ICIs-mediated side effects involves T-lymphocyte-driven inflammation and direct cytotoxicity in many tissues, such as skin, intestine, lungs, liver, endocrine organs and cardiovascular system (4, 5). ICIs mediated cardiotoxicities are rare but can affect anticancer regimens and quality of life in cancer patients (6). Cardiovascular complications in cancer patients treated with ICIs include fatal myocarditis, vasculitis, arrhythmia, fibrosis and heart failure (7, 8). In different types of cancer, different ICIs may show different cardiotoxicity spectra (9). The incidence of ICIs-related cardiovascular events ranged from 0.15 to 10%. For example, in melanomas, PD-1/PD-L1 inhibitor use was closely related to high blood pressure and myocarditis (8, 9). Lung cancer patients, commonly experienced acute coronary syndrome, arrhythmia and heart failure (9). The most common cardiotoxic events after nivolumab and pembrolizumab therapy in lung cancer patients are arrhythmia, cardiac-related chest pain, cardiomyopathy, and myopericardial diseases. Moreover, renal cell carcinomas patients treated with nivolumab associated to ipilimumab frequently experienced hypertension (9); patients with urothelial carcinoma treated with atezolizumab had frequently hypertension and arrhythmia. Therefore, a complex interaction between cancer-related and immune-related factors plays a key role in pathogenesis of cardiovascular toxicities.

Known mechanisms of ICIs-induced cardiotoxicity involves immune-infiltration of CD3 +, CD4 + and CD8 + T lymphocytes in myocardial tissues that can attack cardiomyocytes or endothelial cells leading to metabolic failure and reduced cell viability (10, 11). Notably, ICIs can induce a pro-inflammatory phenotype in cardiac and vascular tissues therefore the identification of new players of cardiotoxicity mediated by short-term ICIs therapy still need further attention. Recent consensus statements highlights on the importance of new predictive biomarkers of ICIs-mediated cardiotoxicity (12, 13). Indeed, echocardiographic biomarkers such as changes in global longitudinal strain (GLS) (12), or myocardial work (MW) (13) or increases in plasma levels of galectin-3 and cytokines (14) are of great interest in clinical cardioncology. Considering that Pembrolizumab and Ipilimumab recognize both human and mice PD-1 and CTLA-4 epitopes (15, 16), we have highlighted on the vascular and myocardial inflammation in female mice through immunohistochemistry and ELISA methods shedding light on potential pathways involved in ICIs cardiotoxicity, including NLRP3, MyD88 and DAMPs.

## Materials and methods

### Cell cultures

Human cardiac cells (HFC cell line; Innoprot, Derio, Spain) were cultured following the manufacturer's instructions (17, 18). Culture medium was supplemented with Fetal Bovine Serum at 10% v/V (FBS, Sigma Aldrich, St. Louis MO, USA), Penicillin at 50 U/ml, Streptomycin at50 μg/mL and L-Glutammine at 1% v/V.

### *In vitro* cytotoxicity assays and cardiac cell lysis

Considering that PD-1 and CTLA-4 are expressed in human cardiomyocytes, as recently analyzed by our group through cell ELISA assays on human fetal cardiomyocytes with anti-PD-1 and anti-CTLA-4 mAbs (19), Ipilimumab and Pembrolizumab were added to HFC cells co-cultured with human lymphocytes. In brief, cells were plated in 96-well flat-bottom plates (at 10,000 cells/well) for 16 h. Human Peripheral Blood Mononuclear Cells (hPBMCs) were added at effector: target ratio 5:1 in the absence or presence of the mAbs (200 nM), and incubated for 24 h at 37°C, as previously described (20). Cells un-incubated or incubated with aspecific antibodies IgG were defined as control. After treatment with antibodies, lymphocytes were removed and adherent cells were washed and counted through trypan blue method. Lymphocytes can be easily removed by collecting the supernatant of co-cultures and by washing the tumor adherent cells as the lymphocytes are non-adherent. Cell survival was expressed as percent of viable cells tested with drugs compared to the untreated ones, used as a negative control. Cardiac cell lysis was determined as described in other recent work (21, 22) through the quantification of released LDH (LDH detection kit, Thermo-Fisher Scientific, Meridian Rd., Rockoford, IL USA), following the manufacturer's instructions.

### ELISA assays on mouse purified proteins and PBMCs

The Enzyme-linked immunosorbent assay (ELISA) was performed on mouse PBMCs and purified recombinant target proteins to test the human-mouse cross-reactivity of the antibodies. Mouse lymphocytes (4 × 10^5^ cells/well) activated with anti-CD3/CD28 beads for 3 days were plated on round-bottom 96-well plates. The purified recombinant human or mouse CTLA-4/Fc or the Fc portion (used as a negative control) were coated on flat bottom plates and blocked with a buffer solution (PBS/milk 5% v/v) for 1 h. The plates were incubated with increasing concentrations of antibodies in a buffer solution (PBS/milk 2.5% v/v) for 2 h. After washing, plates were incubated with HRP-conjugated anti-human IgG (Fab')2 goat monoclonal antibody in a buffer solution (PBS/BSA 3% v/v) for 1 h. The following steps were performed as previously described (23). The Absorbance values at 450 nm were measured by an Envision plate reader (Perkin Elmer, 2102, San Diego, CA, USA).

### Animal studies

Twenty four C57Bl/6 mice (female, 6 weeks/age) were purchased from Harlan, San Pietro al Natisone (Italy). As standard protocol, firstly mice were housed and maintained on a 12 h light-12 h dark cycle in a room with a fixed temperature of 22°C with the appropriate foods water. The experimental protocols were approved by the Ministry of Health with authorization number 1467/17-PR of 13-02-2017, and institutional ethics committees: Organismo preposto al benessere degli animali (OPBA) in accordance with EU Directive 2010/63/EU for animal experiments and Italian D.L.vo 26/2014 law. Briefly, animals were randomly divided into three groups (6 mice per group) as followings:

- Control group: mice daily received normal saline injection (by i.p) every three days for 10 days
- Ipilimumab group: mice received a short therapy with a CTLA-4 blocking agent (Bristol-Myers Squibb, Princeton, New Jersey, US) (15 mg/kg/day by i.p) every three days for 10 days;
- Pembrolizumab group: mice received a short therapy with a PD-1 blocking agent (Merck &amp; Co., Inc., Kenilworth, NJ, USA) (15 mg/kg/day by i.p) every 3 days for 10 days.

Notably, every drug was used in the clinically available formulation. The injected dose of 15 mg/kg mean body-weight was comparable to the uses of ICIs in clinical oncology (16, 17, 24–26); moreover, the dose used is within the range of doses administered intraperitoneally (1–30 mg/kg) in preclinical models for pharmacokinetic studies and anticancer studies with ICIs (15, 27, 28).

### Echocardiographic evaluation of ventricular functions

To assess cardiac functions, the transthoracic echocardiography method was made in mice by using the Vevo 2100 (40-MHz transducer; Visual Sonics, Toronto, ON, Canada) which allows the determination of different cardiac function parameters in anesthetized mice (18, 29, 30). In brief, before (T0), after three days (T3) and at the end of treatments (T10) mice were prepared for cardiac function assessment by previous anesthesia through a solution composed by tiletamine zolazepam (both at 0,09 mg/g of weight) and atropine (at 0,04 mL/g of weight). The left ventricular echocardiography was performed in parasternal long-axis views (with a frame rate corresponding to 233 Hz). Firstly, in M-mode assessment, the left ventricular internal dimensions in diastole and a systole (LVID,d; LVID, s)were calculated from 3 to 5 beats. Fractional shortening and ejection fraction percentage (both in percentage) were determined as described in other recent work (15). Moreover, radial (RS, corresponding to the change in myocardial wall thickness) and longitudinal strain (LS, percent change in length of the ventricle) were calculated on long-axis views following the guide instructions of the Vevo 2100 software (15, 29).

### Myocardial inflammation and DAMPs expression

After treatments, mice were anesthetized as described in section echocardiographic evaluation of ventricular functions and sacrificed via cervical dislocation. Hearts were excised, washed three times in physiological solution (to eliminate blood residues), weighed and divided into two parts through a proper longitudinal cut. One half was used for biochemical studies and the other part to histological analyzes. Firstly, hearts were lysed under ice in a lysis solution consisting of Triton × 100 1% V / v spiked with a protease inhibitor. To promote lysis, tissues were treated with ultrasounds for 5 min. After centrifugation at 4°C at 1,300 rpm for 10 min, the supernatant of the cardiac homogenates were used to quantitative analysis of six biomarkers of cardiac damages and inflammation, such as: myeloid differentiation primary response 88 (MYd-88) expression (through mouse MyD88 ELISA Kit (My Biosource, San Diego, CA, detection range of 78–5,000 pg/ml; sensitivity: 46.9 pg/ml); NOD-, LRR- and pyrin domain-containing protein 3 (NLRP-3) (through mouse NLRP3 ELISA Kit (OKEH05486, Aviva Systems Biology); Fibronectin-EDA, S100/Calgranulin and Galectine-3 [three DAMPs (quantified in cardiac tissues through selective quantitative assay); twelve cytokines and growth factors (IL-1α, IL-1β, IL-2, IL-4, IL-6, IL-10, IL-12, IL17-α, IFN-γ, TNF-α, G-CSF, GM-CSF) through a mouse cytokine Multiplex Assay kit (Qiagen, USA, pg/mg of heart tissue).

### Systemic levels of SDF-1 (CXCL-12), IL-1β, IL-6

For systemic analysis, blood samples were collected in three points: before (T0), after three days (T3) and at the end of treatments (T10) in heparinised tubes and immediately centrifuged at 3,000 rpm for 10 min at 4°C in order to obtain plasma that was collected, frozen, and kept at −80°C until use. Circulating SDF-1 (CXCL-12), IL-1β, IL-6 were quantified in 0,1 mL of plasma through the use of mouse CXCL12/SDF-1 alpha Quantikine ELISA Kit (MCX120, R&D Systems, Minneapolis, MN, USA), mouse IL-1β ELISA kit (BMS6002, Thermo Fisher, Milan, Italy) and mouse IL-6 ELISA kit (KMC0061, Thermo Fisher, Milan, Italy).

### Histology

Blinded histological examination of myocardial tissues were also performed. All selected samples were fixed in formalin and embedded in paraffin. Firstly, tissues were deparaffinized in a solution of xylene and rehydrated through graded alcohols. Antigen retrieval was performed with slides heated in 0.0.1 M edta buffer (pH 8.0.) for 10 min at 110 °C. Slides were rinsed with TBS and treated with a solution at 3 % v/V hydrogen peroxide. Another washing in BSA 5% v/V in PBS was performed as blocking step and an incubation for 12h with a primary antibody (diluted 1:100 in PBS) against mouse NF-kB (Abcam, Cambridge, UK) was performed. Sections were incubated with goat anti- anti-rabbit secondary IgG biotinylated secondary antibody for 0.5 h. Tissue reactivity was determined through the avidin–biotin–peroxydase method (Novocastra, Newcastle, UK) as described in other work (30). After, sections were counterstained with haematoxylin. To determine the structure of the heart, and to evaluate parameters such as hypertrophy, necrosis and fibrosis, the tissues of the heart were incubated with Mayer's hematoxylin for 30 s and washed properly with water (30). Antigen expression was evaluated by one experienced pathologist by using light microscopy. For NF-kB nuclear localization in vascular endothelium of the murine tissues under examination was considered. Immunostaining values were reported as percentage of positive cells in 10 non-overlapping fields by using magnification X400 (31).

## Statistics

Data are presented as means ± standard errors (SE). All data were tested for normality by Shapiro–Wilk. Normally distributed data in two groups were tested with Student's *t*-test, and non-normally distributed data in two groups by Wilcoxon–Mann–Whitney. Normally distributed data in multiple groups were tested by one-way analysis of variance (ANOVA) with Sidak correction. Non-normally distributed data were tested by ANOVA with Holm–Sidak post-testing. Paired data were tested using the paired versions of t-Student.

## Results

### Pembrolizumab and Ipilimumab recognize and bind mouse PD-1 and CTLA-4 and induce cardiotoxic effect in human cardiomyocytes

**Figure 1:**
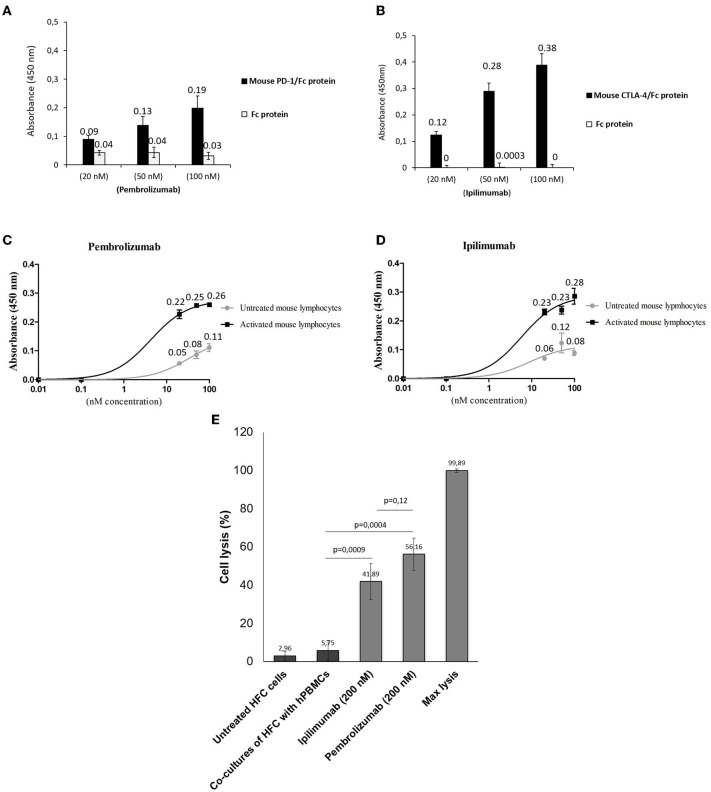
Binding assays of Pembrolizumab and Ipilimumab on mouse PBMCs and on human and mouse purified recombinant target proteins. ELISA assays were performed by using the indicated antibodies at increasing concentrations on mouse (black bars) CTLA-4/Fc or PD-1/Fc **(A,B)**. The binding of antibodies was also tested on the Fc portion (empty bars), used as a negative control. Cell ELISA assays were performed by testing Pembrolizumab **(C)** (*n* = 3) or Ipilimumab **(D)** (*n* = 3) on mouse PBMCs untreated or activated with anti-CD3/CD28 beads. **(E)** LDH assay on the supernatant of co-cultures of HFC and hPBMCs treated as indicated (*n* = 3). Cell lysis was measured as described in the materials and methods section. Error bars depict means ± SD.

We first verified the immunoreactivity of Pembrolizumab and Ipilimumab compared to murine epitopes of PD-1 and CTLA-4, respectively, through ELISA binding assays. As specified in Figure 1, and in line with other recent research (15, 22), Pembrolizumab and Ipilimumab are both able to bind to the mouse targets even though the affinity is lower than that observed for their human counterparts. Cardiotoxicity of Pembrolizumab and Ipilimumab in human *in vitro* models of co-cultures of hPBMC and human cardiomyocytes. To confirm in a human-like environment the results obtained in mouse models on the cardiotoxic effects of the immunomodulatory mAbs, we tested their effects in *in vitro* human models based on co-cultures of human cardiomyocytes (HFC) and lymphocytes. As shown in Figure 1, three mAbs induced a significant cardiac cell lysis (up to 50% for Pembrolizumab), thus indicating that they can indeed activate immune responses against cardiac cells.

### ICI treatment promotes DAMPs and pro-inflammatory cytokine production in co-cultures of hPBMCs and human cardiomyocytes

**Figure 2:**
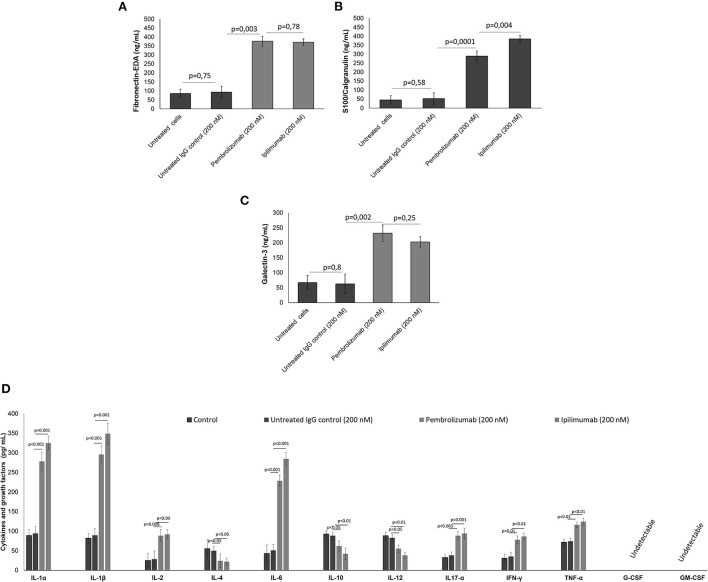
**(A)** DAMPs, including fibronectin, **(B)** S100 calgranulin, **(C)** galectin-3, and **(D)** cytokines and growth factors produced by cardiomyocytes in the supernatant of co-cultures of cardiomyocytes with hPBMC. The cytokines IL-1α, IL-1β, IL-2, IL-4, IL-6, IL-10, IL-12, IL17-α, IFN-γ, TNF-α, G-CSF, GM-CSF (*n* = 3) were quantified by ELISA assays. Error bars depict means ± SD.

It was verified if ICIs could affect the production of DAMPs, pro-inflammatory cytokines, chemokines, and growth factors in co-cultures of human cardiomyocytes with human peripheral blood mononuclear cells (hPBMCs) by analyzing the supernatant by ELISA assays. Firstly, in a similar fashion to the *in vivo* findings, all ICIs increased the production of Fibronectin-EDA (Figure 2A), S199/Calgranulin (Figure 2B) and Galectine-3 (Figure 2C) compared to untreated cells. However, the analysis of cytokines secretion indicated that only IL-1α, IL-1β, IL-6, and TNF-α were significantly increased after incubation with ICIs (Figure 2D).

### Short-term ICI therapy reduces radial/longitudinal strain and ejection fraction

**Figure 3:**
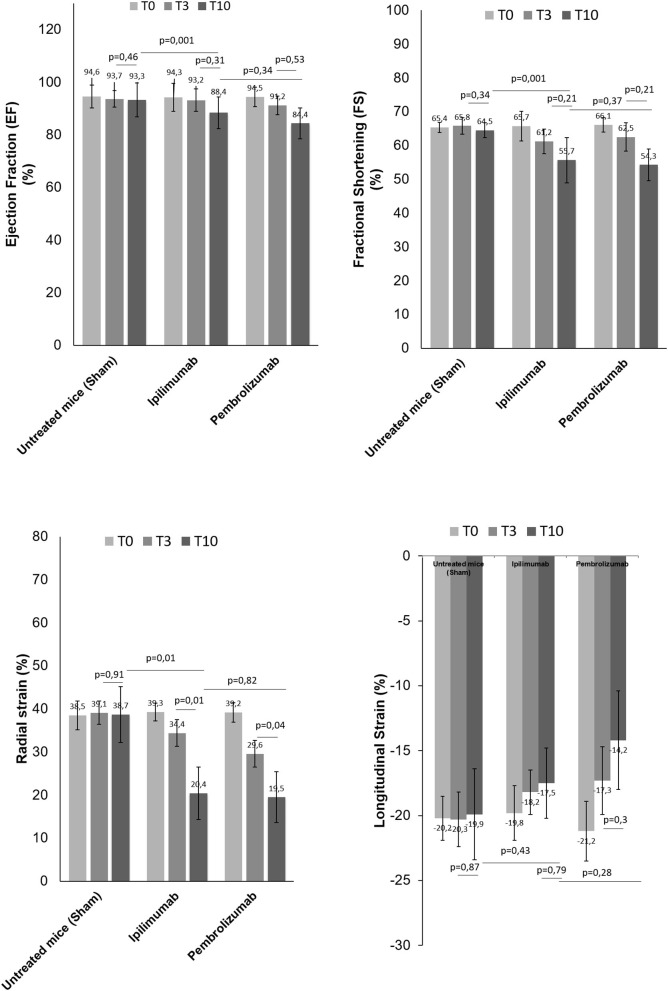
Cardiac function studies. Before (T0), after 3 days (T3) and at the end of treatments (T10) with saline or Ipilimumab or Pembrolizumab (15 mg/kg/die), cardiac functions studies were performed. Ejection fraction (%) (*n* = 6), fractional shortening (%) (*n* = 6), radial (*n* = 6) and longitudinal strain (%) (*n* = 6) were analyzed through VEVO 2100 echocardiography. Error bars depict means ± SD.

We determined the cardiotoxic effects of PD-1 and CTLA-4 blocking agents in C57Bl/6 mice through the study of FS, EF, RS, and LS by using two-dimensional echocardiography (Vevo Strain 2100, Fujifilm). Analysis of EF and FS indicated that short term of ICIs therapies significantly reduces the cardiac function (Figure 3). No differences were seen between groups. Instead, more significant reductions were observed for radial and longitudinal strain in ICIs groups vs. control. Again, no differences between the ICIs were seen (Figure 3).

### Short-term ICI therapy increases vascular expression of nuclear factor kappa-light-chain-enhancer of activated B cells (NF-kB)

**Figure 4:**
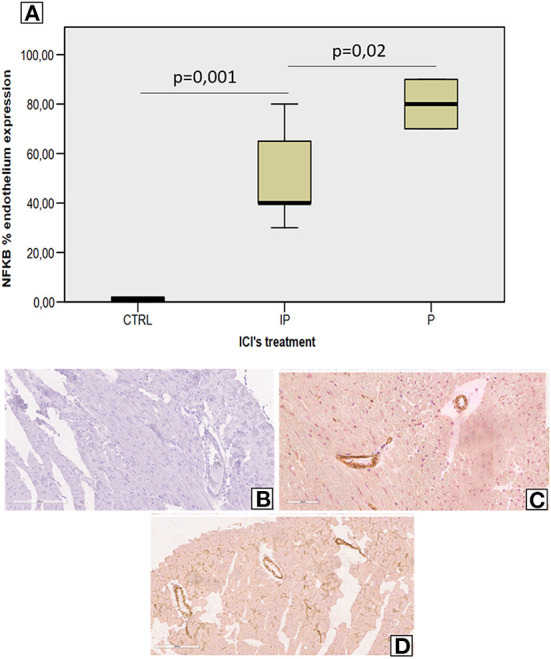
Endothelial NF-kB expression in myocardial tissues of mice treated with ICIs **(A**) Quantitative analysis of NF-kB endothelial expression (%) in mice untreated or treated with ICIs (*n* = 6). Vascular expression of NF-kB in mice untreated **(B)** or treated with Ipilimumab **(C)** or Pembrolizumab **(D)**.

Analysis of vascular and myocardial NF-kB expression shows that short-term anti-CTLA-4 or anti-PD-1 treatment had differential effects (Figure 4). However, first, both ICIs increased significantly the vascular inflammation compared to untreated mice but the higher NF-kB expression was seen in Pembrolizumab compared to Ipilimumab group (Figure 4A). Histological characterization of the myocardial tissue phenotype confirms no detectable vascular NF-kB expression in untreated mice (Figure 4B) but a strong vascular expression was seen in ICIs groups (see arrows in Figures 2C,D).

### Short-term Pembrolizumab therapy increases cardiac hypertrophy

**Figure 5:**
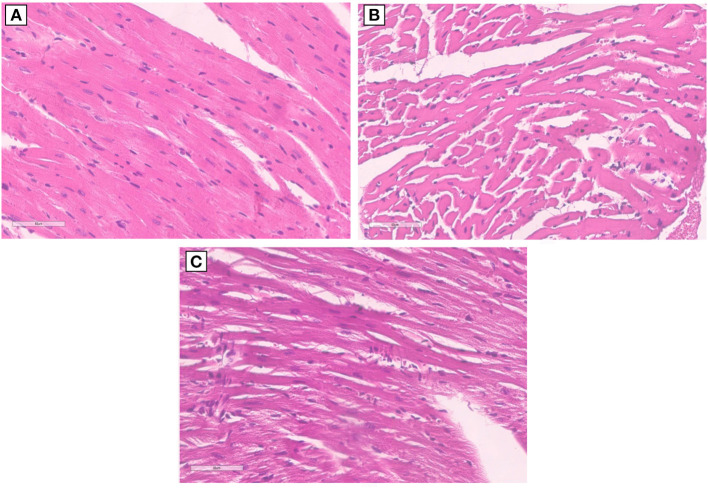
Hypertrophy analysis in myocardial tissues of mice treated with ICIs. **(A)** Hematoxylin-eosin staining of longitudinal sections with no hypertrophy in control untreated mice (x40); **(B)** hematoxylin-eosin staining of longitudinal sections with no hypertrophy in untreated mice and mice treated with Ipilimumab (×40); **(C)** hematoxylin-eosin staining of longitudinal sections highlights the development of marked hypertrophy in mice treated with Pembrolizumab (×40).

Morphological characterization of the myocardial tissue phenotype in mice after short-term anti-CTLA-4 or anti-PD-1 treatment clearly indicates no cardiac hypertrophy with the exception of Pembrolizumab (Figure 5). Compared to control (sham), ematoxylin-eosin staining of longitudinal sections of mice treated with Ipilimumab did not show any significant hypertrophy; cardiomyocytes have a linear and homogeneous longitudinal aspect, the nucleus is not pycnotic and has a regular size and shape, and there are no cytoplasmic vacuoles. On the other hand, the cardiac longitudinal section of mice treated with Pembrolizumab shows a significant hypertrophy with evident increases in the cytoplasmic volume and an irregular course of the cardiomyocytes (Figure 5).

### Short-term ICI therapy increases cardiac fibrosis and myocardial expression of MMP-9, Galectin-3, and pro-collagen-α1

**Figure 6:**
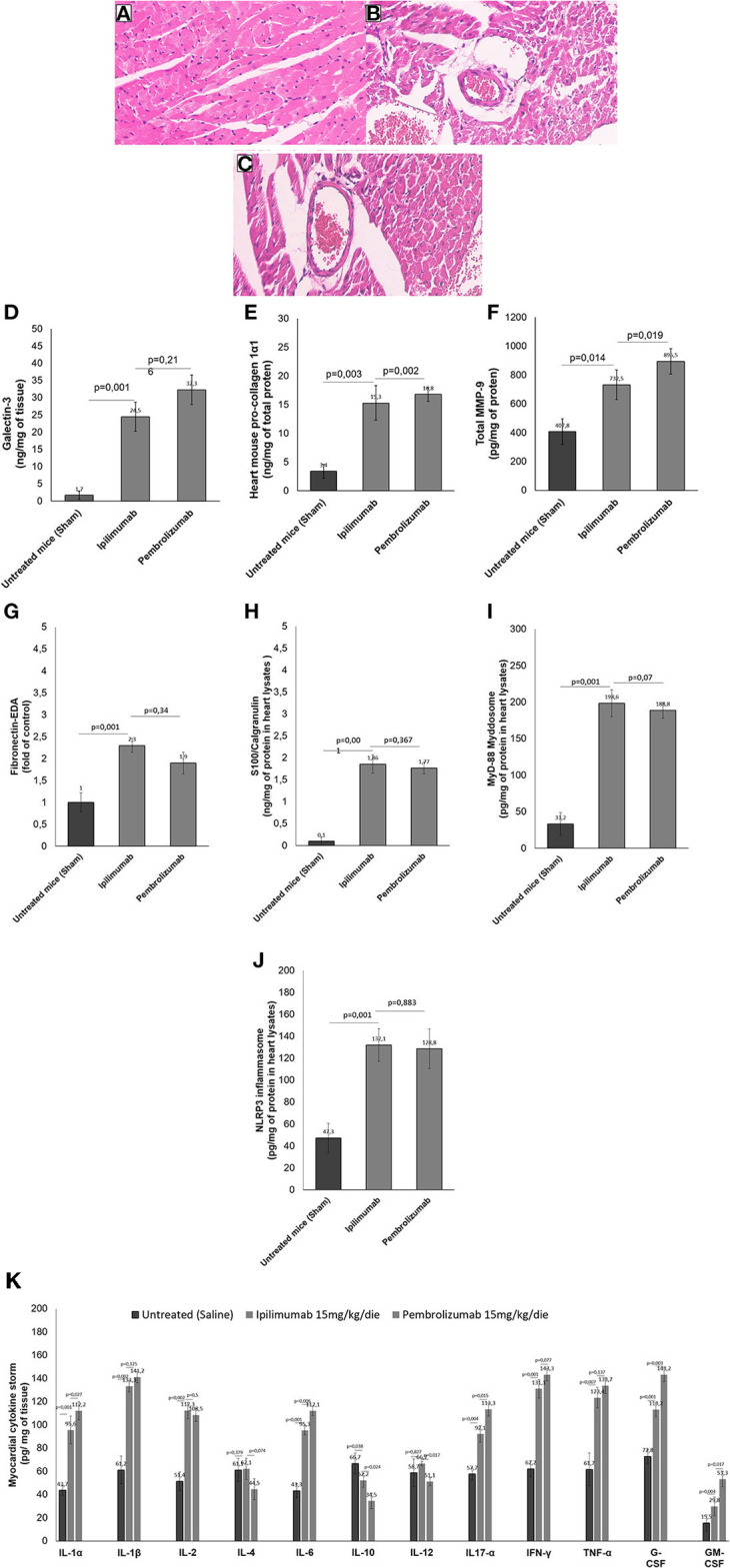
Histological analysis of the hearts of mice untreated or treated with ICIs. **(A)** Hematoxylin-eosin staining of longitudinal sections in control untreated mice (x40); **(B)** hematoxylin-eosin staining of longitudinal sections in mice treated with Ipilimumab (×40); **(C)** hematoxylin-eosin staining of longitudinal sections with fibrosis in mice treated with Pembrolizumab (×40). **(D)** Cardiac expression of galectin-3 (ng/mg of tissue), **(E)** mouse pro-collagen 1-α (ng/mg of total proten), **(F)** MMP-9 (pg/mg of protein), **(G)** Fibronectin EDA (fold of control), **(H)** S100 Calgranulin (ng/mg of protein in myocardial tissue lysate), **(I)** MyD-88 (pg/mg of protein in myocardial tissue lysate) and **(J)** NLRP-3 (pg/mg of protein in myocardial tissue lysate) after short-term treatment with ICIs. In **(K)**, Twelve cytokines (IL-1α, IL-1β, IL-2, IL-4, IL-6, IL-10, IL-12, IL17-α, IFN-γ, TNF-α, G-CSF, GM-CSF) were analyzed in heart lysates and reported as pg of cytokine normalized for mg of tissue. Error bars depict means ± SD (*n* = 6).

Histological characterization of the myocardial tissue phenotype in mice after short-term anti-CTLA-4 or anti-PD-1 treatment showed that ICIs increased cardiac fibrosis compared to untreated mice (Figures 6A–C). In control group (Figure 6A) hematoxylin-eosin staining of longitudinal sections evidenced the absence of fibrosis, whereas treatment with Ipilimumab or Pembrolizumab (Figures 6B,C) increased drastically the fibrotic phenotype. Myocardial expression of galectin-3 (Figure 6D), mouse pro-collagen 1-α (Figure 6E) and MMP-9 (Figure 6F), biomarkers of fibrosis, corroborates these findings: galectin-3 is almost undetectable in control group while it is drastically increased after treatment with all ICIs (1.7 ± 1.2 vs. 24.5 ± 4.2 vs. 32.3 ± 4.3 ng/mg of tissue for control, Ipilimumab and Pembrolizumab, respectively; *p* < 0.001 for control vs. ICIs). Similar results were seen for procollagen 1α1 (3.4 ±1.2 vs. 15.3 ± 3.1 vs. 16.8 ± 1.2 ng/mg of protein for control, Ipilimumab and Pembrolizumab, respectively; *p* < 0.005 for control vs. ICIs) and MMP-9 (407.8 ± 89.6 vs. 732.5 ± 102.2 vs. 895.5 ± 88.6 pg/mg of protein for control, Ipilimumab and Pembrolizumab, respectively; *p* < 0.05 for control vs. ICIs).

### ICI therapy promotes DAMPs production, NLRP3, and MyD88 expression in myocardial tissues

We investigated on cardiac markers of inflammation and cell damages (DAMPs). Compared to untreated mice, ICIs increased significantly Fibronectin-EDA expression (Figure 6G) in heart lysates (2.3 ± 0.16 for Ipilimumab and 2.1 ± 0.25 for Pembrolizumab; *p* < 0.001 for control vs. ICIs). Similarly another DAMP, called S100 Calgranulin (Figure 6H) was significantly enhanced by ICIs treatment (1.86 ± 0.21 for Ipilimumab and 1.96 ± 0.24 ng/mg of protein for Pembrolizumab; *p* < 0.001 for control vs. ICIs); no significant differences between ICIs were seen. Inflammasome and myddosome complex stimulates DAMPs, therefore, myocardial expression of NLRP type 3 and MyD type 88 were analyzed. A drastic increase in MyD-88 expression (Figure 6I) (33.2 pg/mg of protein ± 15.6 for untreated mice:198.6 ± 18.3 for Ipilimumab; 217.5 ± 17.4 for Pembrolizumab; *p* < 0.005 for ICIs vs. control). The same behavior was seen for NLRP3 expression after treatment with all ICIs (47.3 pg/mg of protein ± 13.5 for untreated mice: 132.1 ±15.1 for Ipilimumab; 126.6 ± 11.2 for Pembrolizumab; *p* < 0.005 for ICIs vs. control) (Figure 6J).

### ICI therapy increases pro-inflammatory cytokine expression in myocardial tissues

Cytokines and chemokines are drivers of anticancer drug-induced cardiotoxicity, heart failure and myocarditis (32). Therefore, cytokines and chemokines in heart tissue of Ipilimumab or Pembrolizumab-treated female C57Bl6 mice were quantified (Figure 6K). Firstly, the family of IL-1 cytokines (IL-1α and IL-1β), increased in all ICIs treated group with respect to untreated mice (*p* < 0.001). IL-2 levels were also increased in ICIs group, highlighting immune-related reactions in myocardial tissue. Anti-inflammatory cytokines levels (IL-4 and IL-10) were reduced in ICIs group vs. untreated mice. Other pro-inflammatory cytokines (IL-6, IL17-α, IFN-γ, and TNF-α) were also increased in myocardial tissues of ICIs-treated mice vs. saline-treated groups. Levels of growth factors involved in heart failure and hypertrophy (G-CSF and GM-CSF) were also increased in ICIs groups (Figure 6K).

### ICI therapy increases systemic levels of SDF-1 (CXCL12), inteleukin-1β, and interleukin-6

**Figure 7:**
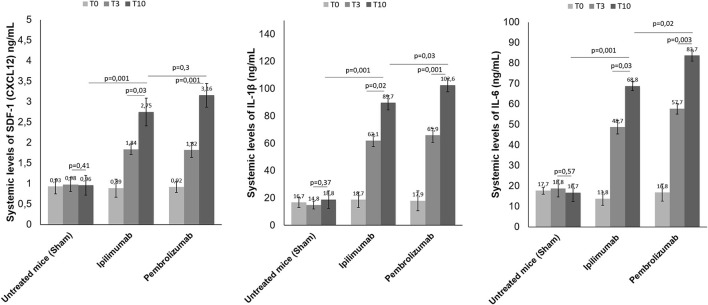
Effects of ICI treatment on plasma levels of SDF-1 (CXCL-12), Interleukin 1-β and Interleukin-6. Female C57/Bl6 mice were treated daily with anti-CTLA-4 or anti-PD-1 antibodies (*n* = 6) or left untreated (*n* = 6) for 10 days. Before (T0), after three days (T3) and at the end of treatments (T10), SDF-1 (*n* = 6), Interleukin 1-β (*n* = 6) and Interleukin-6 (*n* = 6) were quantified in plasma. Levels were reported in ng/ml of plasma.

High plasma levels of Stromal Cell-Derived Factor 1 (SDF-1), IL-1β and IL-6 were associated to cardiac inflammation, heart failure and cardiovascular mortality (33). We analyzed if short term ICIs therapy could affect SDF-1, IL-1 β and IL-6 levels in plasma of C57/Bl6 mice (Figure 7). Before (T0), after three days (T3) and at the end of treatment (T10), systemic levels of all biomarkers were significantly increased with respect to control saline-treated mice (*p* < 0.05). For example, after 10 days of therapy, SDF-1 levels were 0.96 ng/ml ±0.24 for untreated mice; 2.75± 0.34 for Ipilimumab and 3.16 ± 0.29 for Pembrolizumab (*p* < 0.005 for ICIs vs. control). The same behavior was seen for IL-1 β and IL-6, indicating a systemic inflammation even after 3 days of treatment with ICIs (Figure 7).

## Discussion

**Figure 8:**
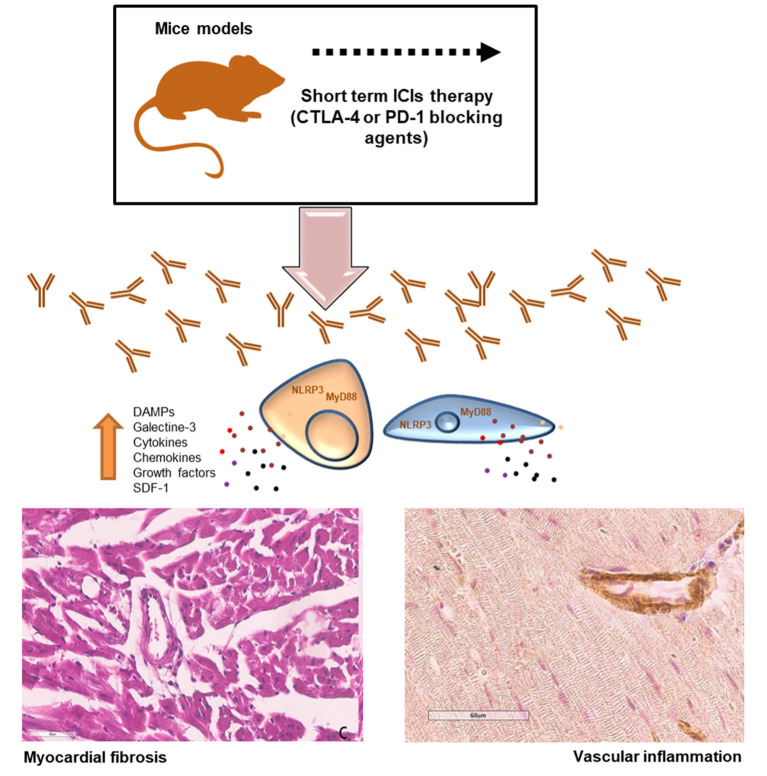
Short-term ICI Therapy increases DAMPs, NLRP3 and MyD-88 mediated fibrosis and vascular inflammation. Short-term treatment with immune checkpoint inhibitors (ICIs) induced significant increases in DAMPs, galectine-3, cytokines and chemokines through NLRP3 and MyD88 pathways inducing myocardial hypertrophy, fibrosis and strong vascular inflammation.

The current study aimed to evaluate the pro-inflammatory effects of short-term immune checkpoint inhibitors (ICIs) treatment in myocardial and vascular tissues in preclinical models (34, 35) (Figure 8). Notably, the use of ICIs in clinical oncology found significant clinical benefits in cancer patients, however, a wide spectrum of side effects are being seen (called irAEs): rash (maculopapular, lichenoid), diarrhea, colitis, mucositis, hypo or hyper thyroidism, hepatitis, inflammatory arthritis, myalgia (36). Endocrinopathies and inflammatory pathologies induced by PD-1 / PDL-1 or CTLA-4 blocking agents are frequently reported in both monotherapy and combinatorial therapies. Clinical studies report both short-term and long-term side effects in cancer patients and the mechanisms are not yet well-known (37). There are both immune-mediated and non-immune-mediated mechanisms involved in irAEs. A recent meta-analysis reports a high incidence of myocarditis (about 11 times higher than in other therapies) which reaches a mortality rate of about 50% in case of combination therapies (PD1 / PDL-1 associated to CTLA-4 blocking agents) (38, 39). Furthermore, ICIs have recently been shown to accelerate the process of atherosclerosis in both preclinical and clinical study models. Even just a short treatment with ICIs increases the inflammatory state in the vascular endothelium by accelerating the atherosclerotic process (39). Other recent studies show that short ICSi treatments can cause arrhythmias, Takotsubo syndrome and inflammatory vascular events (40, 41). Current data regarding ICIs- associated pericardial involvement are limited, but case-reports include pericarditis, pericardial effusion (42, 43). Notably, a deep knowledge of ICIs-induced myocardial injuries is needed. Immune cells uptake and infiltration in myocardial tissue were always seen in human histological studies (CD4, CD8 T cells and macrophages). Immune-related side effects involves several chemokines like CXCR 10, 9 and 3, high levels of granzyme B especially in myocardial tissue (44). Our data suggest that NLRP3 and MyD88 pathways could contribute to the increased vascular and myocardial inflammation of anti-CTLA-4/anti-PD-1 treatments. NLRP3 drives cytosolic damages, hypertrophy and inflammation through cytokines and overproduction of hs-CRP.

As described in Figure 6 and summarized in Figure 8, our data confirms that ICIs increases DAMPs in cardiomyocytes and myocardial tissue of mice models. It has been described that in patients with unstable angina or with AMI, endogenous DAMPs like Fibronectin-EDA, S100/Calgranulin, Galectine-3 are released from damaged cardiac cells and signal through TLR receptors. There is also emerging evidence for the involvement of Toll-like receptors type-9 in heart failure, which can be activated by endogenous DAMPs, including mitochondrial DNA, to modulate the progression of the disease (45, 46). The increases in DAMPs levels after short-term ICIs treatment in mice indicates myocardial injuries (46). Whether long-term ICI therapy affects myocardial stress and vascular inflammation is unknown. Nevertheless, our data suggest that even short-term ICIs therapy induces vascular inflammation, fibrosis and levels of myocardial NLRP3 and MyD88 (Figure 8). Whether ICIs- induced inflammation persist after cessation of the therapy and how they affect myocardial work in the long-term is not currently known (47).

This work has several limitations: firstly, the use of a tumor-free mice model. As cardiovascular diseases and cancer share many pathophysiological pathways, including inflammation, the use of a tumor-bearing model would have increased the translational potential of our study. Moreover, there are clinical evidences that combination ICIs-therapy exerts the most frequent and pro-inflammatory cardiac and endocrine side effects compared to monotherapies (48) therefore, further cardiotoxic studies in preclinical models will be performed after the combination of anti-CTLA4 and anti-PD-1/PD-L1.

Another limitation is based on the short period of treatment with ICIs without a longer follow up. In real world clinical experience, cancer patients experienced ICIs-mediated side effects also many months after therapy cessation (47, 49). These effects could be also partially related to endocrine changes due to PD-1/CTLA-4 blocking pathways that exerts a detrimental cardiotoxic effects in these patients. Therefore, further preclinical studies on long-term cardiovascular side effects after ICIs therapy will be performed.

Another methodological limitation of this work is the absence of a more proper control group in animal studies based on the administration of IgG control antibody, however, as reported in cellular experiments (Figure 8), any changes in pro-inflammatory and cell dead markers were seen after incubation with control IgG. Moreover, it is plausible that the intraperitoneal administration of nonspecific IgG as a control does not change neither cardiac functions nor cardiac and systemic inflammatory status in mouse models as confirmed by recent similar research papers (50, 51).

In conclusion, cardiotoxicity, although rare, is a clinically relevant problem in cancer patients undergoing ICIs. Long-term side effects of ICIs are reported, however, some biochemical changes may occur even a few days after treatment with ICIs, as specified in this work. The involvement of NLRP3 MyD-88 and some DAMPs in ICIs-associated cardiovascular disease was seen. Relevant histological effects such as cardiac endothelial inflammation and overexpression of pro-fibrotic and pro-inflammatory cytokines is a non-negligible fact that deserves further investigation. The increase in systemic levels of SDF-1, IL-1β and il-6 indicates systemic pro-inflammatory effects induced by ICIs that can directly and indirectly increase the risk of myocarditis, however more detailed studies on the mechanisms of systemic and direct cardiac toxicity will have to be carried out. Of note, considering that PD-1 and CTLA-4 blocking agents recognize the murine epitope with lower affinity than the human epitope, the effects observed in this work could also be underestimated (12) consequently, short and long term clinical studies during ICIs deserve urgent investigation. Moreover, the results of this study warrant further preclinical cardioprotective trials with anti-cytokine (52, 53), anti-NLRP3 (54–56) or anti-MyD88 (57, 58) therapies in primary or secondary prevention of ICIs-related cardiotoxicity.

This study suggests that short-term ICI therapy affects myocardial and vascular inflammation through DAMPs and cytokines through NLRP-3 and MyD-88 related pathways (Figure 8). It is plausible that ICIs exerts both systemic and cardiac toxicities through the activation of cytokines cascades that exacerbate the inflammatory damages in cardiomyocytes. These results are in line with another recent work (59) demonstrating pro-atherosclerotic effects of short-ICIs therapy in mice models. Clinical studies are required to elucidate the effects of ICIs on myocardial and vascular inflammation and confirms the role of NLRP3 and Myd88 in progression of ICIs-mediated cardiovascular diseases.

## Data availability statement

The data presented in the study are deposited in the Zenodo repository, accession number https://zenodo.org/record/7040431#.YyMLcYrP1D9.

## Ethics statement

The animal study was reviewed and approved by the experimental protocols, in accordance with EU Directive 2010/63/EU for animal experiments and Italian D.L.vo 26/2014 law, were approved by the Ministry of Health with authorization number 1467/17-PR of 13-02-2017, and institutional ethics committees: Organismo preposto al benessere degli animali (OPBA).

## Author contributions

VQ, SB, MP, AD, CC, GG, AB, GP, and AL performed the experiments and analyzed the data. AP, IB, AI, and MC provided technical assistance. VQ, CD, and NM designed the study and drafted the manuscript. All authors read and approved the final manuscript.

## Funding

This work was funded by a “Ricerca Corrente” grant from the Italian Ministry of Health titled “Cardiotossicità da chemioterapie, targeted therapies e immunoterapie, diagnosi precoce e cardioprotezione, Ricerca preclinica e clinica”.

## Conflict of interest

Authors MP and CD were employed by Ceinge-Biotecnologie Avanzate. The remaining authors declare that the research was conducted in the absence of any commercial or financial relationships that could be construed as a potential conflict of interest.

## Publisher's note

All claims expressed in this article are solely those of the authors and do not necessarily represent those of their affiliated organizations, or those of the publisher, the editors and the reviewers. Any product that may be evaluated in this article, or claim that may be made by its manufacturer, is not guaranteed or endorsed by the publisher.
