# Corrigendum: Immune checkpoint inhibitor therapy increases systemic SDF-1, cardiac DAMPs Fibronectin-EDA, S100/Calgranulin, galectine-3, and NLRP3-MyD88-chemokine pathways

**DOI:** 10.3389/fcvm.2023.1129873

**Published:** 2023-01-19

**Authors:** Vincenzo Quagliariello, Margherita Passariello, Annabella Di Mauro, Ciro Cipullo, Andrea Paccone, Antonio Barbieri, Giuseppe Palma, Antonio Luciano, Simona Buccolo, Irma Bisceglia, Maria Laura Canale, Giuseppina Gallucci, Alessandro Inno, Claudia De Lorenzo, Nicola Maurea

**Affiliations:** ^1^Division of Cardiology, Istituto Nazionale Tumori- Istituto di Ricovero e Cura a Carattere Scientifico (IRCCS)- Fondazione G. Pascale, Naples, Italy; ^2^Department of Molecular Medicine and Medical Biotechnology, University of Naples “Federico II”, Naples, Italy; ^3^Ceinge-Biotecnologie Avanzate s.c.a.r.l., Naples, Italy; ^4^Pathology Unit, Istituto Nazionale Tumori- Istituto di Ricovero e Cura a Carattere Scientifico (IRCCS)- Fondazione G. Pascale, Naples, Italy; ^5^Animal Facility, Istituto Nazionale Tumori- Istituto di Ricovero e Cura a Carattere Scientifico (IRCCS)- Fondazione G. Pascale, Naples, Italy; ^6^Servizi Cardiologici Integrati, Dipartimento Cardio-Toraco-Vascolare, Azienda Ospedaliera San Camillo Forlanini, Rome, Italy; ^7^U.O.C. Cardiologia, Ospedale Versilia, Lido di Camaiore (LU), Camaiore, Italy; ^8^Cardiologia, Centro di Riferimento Oncologico della Basilicata (CROB) - Istituto di Ricovero e Cura a Carattere Scientifico (IRCCS), Rionero in Vulture, Italy; ^9^Medical Oncology, Istituto di Ricovero e Cura a Carattere Scientifico (IRCCS) Ospedale Sacro Cuore Don Calabria, Negrar, Italy

**Keywords:** immunotherapy, biomarkers, preclinical study, mechanisms, inflammation, interleukin

In the published article, there was an error in the **Data Availability** statement. The original statement incorrectly read: “The raw data supporting the conclusions of this article will be made available by the authors, without undue reservation.” The correct **Data Availability** statement appears below:

“The data presented in the study are deposited in the Zenodo repository, accession number https://zenodo.org/record/7040431#.YyMLcYrP1D9.”

In the published article, there was an error in the **Funding** statement. The original statement incorrectly read: “This work was funded by a Ricerca Corrente grant from the Italian Ministry of Health.” The correct **Funding** statement appears below:

“This work was funded by a “Ricerca Corrente” grant from the Italian Ministry of Health titled “Cardiotossicità da chemioterapie, targeted therapies e immunoterapie, diagnosi precoce e cardioprotezione, Ricerca preclinica e clinica”.

The authors apologize for these errors and state that they do not change the scientific conclusions of the article in any way. The original article has been updated.

